# Vaccination with *Acinetobacter baumannii* adhesin Abp2D provides protection against catheter-associated urinary tract infection

**DOI:** 10.21203/rs.3.rs-3213777/v1

**Published:** 2023-08-10

**Authors:** Morgan R. Timm, Kevin O. Tamadonfar, Taylor M. Nye, Jerome S. Pinkner, Karen W. Dodson, Ali H. Ellebedy, Scott J. Hultgren

**Affiliations:** 1 Department of Molecular Microbiology and Center for Women’s Infectious Disease Research, Washington University School of Medicine, St. Louis, MO, USA.; 2 Department of Pathology and Immunology, Center for Vaccines and Immunity to Microbial Pathogens, and The Andrew M. and Jane M. Bursky Center for Human Immunology and Immunotherapy Programs, Washington University School of Medicine, St Louis, MO, USA.

## Abstract

Catheter-associated urinary tract infections (CAUTIs) contribute greatly to the burden of healthcare associated infections. *Acinetobacter baumannii* is a Gram-negative bacterium with high levels of antibiotic resistance that is of increasing concern as a CAUTI pathogen. *A. baumannii* expresses fibrinogen-binding adhesins (Abp1D and Abp2D) that mediate colonization and biofilm formation on catheters, which become coated with fibrinogen upon insertion. We developed a protein subunit vaccine against Abp1D_RBD_ and Abp2D_RBD_ and showed that vaccination significantly reduced bladder bacterial titers in a mouse model of CAUTI. We then determined that immunity to Abp2D_RBD_ alone was sufficient for protection. Mechanistically, we defined the B cell response to Abp2D_RBD_ vaccination and demonstrated that immunity was transferrable to naïve mice through passive immunization with Abp2D_RBD_-immune sera. This work represents a novel strategy in the prevention of *A. baumannii* CAUTI and has an important role to play in the global fight against antimicrobial resistance.

## Introduction

The U.S. Centers for Disease Control and Prevention (CDC) estimates that 1 out of every 31 hospitalized patients in the U.S. will acquire at least one healthcare-associated infection (HAI) over the course of their care.^1^ HAIs are problematic because they lead to an increased burden of morbidity and mortality for patients, cost the US healthcare system an estimated $35.7 billion per year, and drive antibiotic overuse that contributes to the development of antimicrobial resistance.^2^ One of the most common types of HAIs are catheter-associated urinary tract infections (CAUTIs). The risk of CAUTI increases by 3-7% with each subsequent day of catheterization and approaches 100% in patients catheterized for more than 30 days.^3,4^ More than 30 million Foley (urinary) catheters are used annually in the United States.^5,6^ Because catheter use is so ubiquitous, CAUTIs make up nearly 40% of all HAIs in the US each year.^7^ Unfortunately, despite concerted infection mitigation efforts by public health agencies and healthcare facilities, the rate of CAUTIs continues to rise and increased by 5% from 2019-2021 in the United States.^1^

*Acinetobacter baumannii* is a Gram-negative bacterium implicated in multiple types of HAIs. While it is best known for causing ventilator-associated pneumonia, it is increasingly recognized as an important CAUTI pathogen.^8^ Several single-center studies have identified *A. baumannii* as a leading cause of CAUTI in their facilities.^8,9^ Indeed, recent studies have demonstrated that 17% of published *A. baumannii* isolates originated in the urinary tract and up to 2% of the healthy population may exhibit *A. baumannii* asymptomatic bacteriuria.^10,11^ At the same time, the level of antimicrobial resistance identified in *A. baumannii* isolates is on the rise, with most isolates resistant to at least one antibiotic class and many isolates displaying multi-drug resistance.^12^ As a result, the CDC and World Health Organization have classified carbapenem-resistant *A. baumannii* as an “urgent threat,” which is the highest threat level.^13^ Together, the prevalence of *A. baumannii* coupled with its multi-drug resistance profile have emphasized the critical need for antibiotic-sparing therapeutics for *A. baumannii* CAUTI.

Many Gram-negative bacteria produce hair-like proteinaceous fibers called pili, tipped by specialized adhesins that recognize receptors with stereochemical specificity to determine host and tissue tropisms. The class of pili most implicated in host-pathogen interactions are the chaperone-usher pathway (CUP) pili.^14^ CUP adhesins are two-domain proteins with an amino terminal receptor binding domain (RBD) and a pilin domain that links the adhesin to the pilus rod. Importantly, new therapies that neutralize the function of the receptor binding domain of CUP pilus adhesins in a variety of pathogens have been successful in preclinical models and early human clinical trials.^15-24^ Our recent studies of *A. baumannii* CAUTI pathogenesis have revealed that *A. baumannii* CUP adhesins are critical in catheter colonization and thus may represent promising drug targets.^8,25^ When a urinary catheter is inserted into the bladder, it induces inflammation and leads to deposition of host proteins, such as fibrinogen, onto the surface of the implant.^23,24,26-28^ *A. baumannii* have evolved two CUP pili, Abp1 and Abp2, tipped with fibrinogen binding adhesins Abp1D and Abp2D respectively. The majority of published *A. baumannii* genomes encode one or both of these pilus operons.^25^ Both of these adhesins have been shown to bind fibrinogen and to be critical in a mouse model of CAUTI.^25^ Therefore, we hypothesized that targeting the Abp1D/Abp2D adhesins with an adhesin-based vaccine, comprised of their receptor binding domains Abp1D_RBD_ and Abp2D_RBD_, would prevent *A. baumannii* CAUTI pathogenesis.

Here, we present evidence that vaccination with recombinant Abp2D_RBD_ provides protection from *A. baumannii* CAUTI in a mouse model. Immunity conferred by a previous *A. baumannii* infection was used to establish a baseline for vaccine performance. We demonstrate that a vaccine formulation containing both adhesins provided protection from *A. baumannii* CAUTI that was superior to natural infection. Further, we show that immunity to Abp2D_RBD_ alone was sufficient for protection. We demonstrate that our Abp2D_RBD_ vaccine generates robust memory B cell and bone marrow plasma cell responses, and that antibody-mediated protection is transferable to naïve mice via passive immunization. This work provides proof-of-concept that an adhesin-based vaccine may be a promising strategy for multidrug-resistant *A. baumannii* CAUTI and could directly contribute to the arsenal of antibiotic-sparing therapeutics needed to meet the urgent threat of antibiotic-resistant *A. baumannii*.

## Results

### Natural infection provides protection against subsequent *A. baumannii* CAUTI infection despite lack of adhesin-specific IgG response.

**Figure 1:**
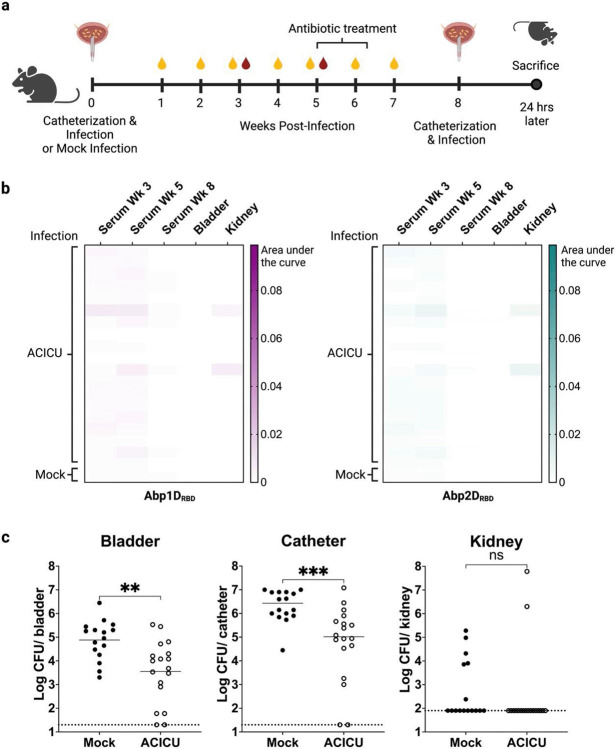
Immunity from prior *A. baumannii* infection. a) 6-7 week old C57/Bl6 mice were catheterized and infected with *A. baumannii* strain ACICU or mock-infected with PBS. Urine was collected to monitor infection status. Mice received 10 days of oral apramycin (1 mg/L) at Week 5 to clear the infection. At week 8, mice were catheterized and challenged with A. baumannii strain ACICU and sacrificed 24 hours post infection. b) Bladder and kidney homogenates and serum from Weeks 3, 5, and 8 were assayed for Abp1D_RBD_- and Abp2D_RBD_-specific IgG by ELISA. Heatmaps were generated by calculating the area under the curve for each serum/tissue sample. c) Bladder, catheter, and kidney titers were enumerated. Dashed lines indicate limit of detection. Mann-Whitney U-test, ***P≤0.0005, **P≤0.005.

We have previously shown that adhesins Abp1D and Abp2D are critical virulence factors for *A. baumannii* CAUTI pathogenesis.^25^ However, prior studies did not examine the immune response to Abp1D/Abp2D during infection, nor did they consider whether a history of infection would provide any degree of protection from a subsequent challenge infection. To investigate this question, we catheterized C57BL/6 mice, infected with *A. baumannii* strain ACICU or mock-infected with PBS, and then treated with apramycin at week 5 to clear the infection (Figure 1a). We then analyzed the serum, bladders, and kidneys of treated mice, termed convalescent mice, for IgG specific to Abp1D_RBD_ and Abp2D_RBD_ to determine if an immune response was elicited against these adhesins. While two individuals developed a low level of Abp1D or Abp2D-specific IgG (AUC ~0.005), most animals did not generate any appreciable antigen-specific IgG response (AUC < 0.002) (Figure 1b). The low levels of antigen specific IgG to the Abp1 and 2 adhesins was not surprising, as this has been observed in other pilus systems.^19^ Each pilus is comprised of thousands of rod subunits tipped by a single adhesin. When mice are infected with whole bacterial cells or immunized with whole pili, the antibody response is skewed towards the much more abundant rod subunit and the response to the adhesin is minimal.^19,23^ That said, CAUTI mice treated with antibiotics and subsequently re-infected displayed a reduction in bladder and catheter bacterial titers of approximately 1 log compared to naïve mice (Figure 1c). The lack of IgG titers suggests that factors other than adhesin-specific IgG, such as epithelial trained immunity or adaptive immunity to other bacterial epitopes, are likely responsible for the protective effect. ^29-33^

### Immunization with *A. baumannii* Abp1/Abp2 adhesins provides protection from CAUTI.

**Figure 2:**
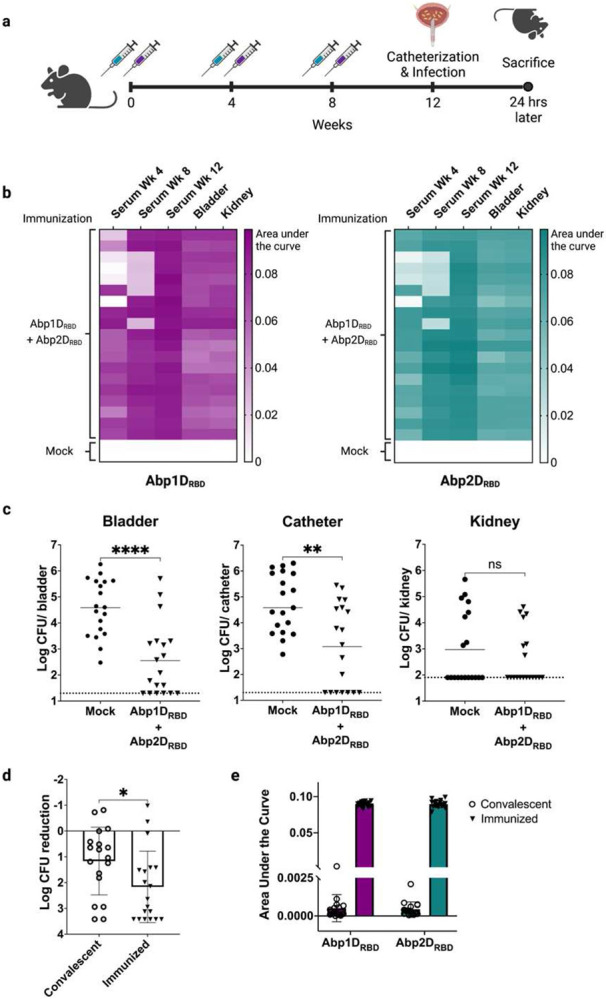
Immunization with Abp1D and Abp2D provides protection from CAUTI. a) 6-7 week old C57/Bl6 mice received 3 adjuvanted doses of 50 ug Abp1D_RBD_ and 50 ug Abp2D_RBD_ or buffer alone (mock). Serum was collected at weeks 4 and 8 prior to immunizations, and at week 12 following sacrifice. Four weeks after the third dose, mice were catheterized and challenged with *A. baumannii* strain ACICU. Mice were sacrificed 24 hours after infection. b) Bladder and kidney homogenates and serum from Week 4, Week 8, and the day of sacrifice were assayed for Abp1D_RBD_ and Abp2D_RBD_-specific IgG by ELISA. Heatmaps were generated by calculating area under the curve for each serum/tissue sample. c) Bacterial titers were enumerated from bladders, catheters, and kidneys. d) Normalized reduction in bacterial titers in the bladders of convalescent and Abp1D_RBD_/Abp2D_RBD_ immunized mice. e) ELISA AUCs of Abp1D_RBD_ and Abp2D_RBD_-specific IgG in serum from convalescent vs. Abp1D_RBD_/Abp2D_RBD_ immunized mice. Mann-Whitney U-test, ***P≤0.0005, **P≤0.005, *P≤0.05.

Mutations in *abp1/abp2* attenuate virulence.^8,25^ Thus, based on work with other adhesin-based vaccines,^19,23,24^ we hypothesized that immunization with the *A. baumannii* adhesins, Abp1D and Abp2D, might confer increased protection relative to natural immunity. We purified the Abp1D and Abp2D receptor binding domains (RBDs) as previously described^25^ and used the proteins to immunize C57BL/6 mice. Four weeks after the final immunization, mice were catheterized and infected with strain ACICU (Figure 2a). Since the efficacy of most vaccines depends on eliciting a strong antigen-specific IgG response, we collected serum at: i) week 4 (prior to 2^nd^ immunization); ii) week 8 (prior to 3^rd^ immunization); and iii) week 12 (at time of sacrifice), to test for Abp1D_RBD_- and Abp2D_RBD_-specific IgG. We also tested bladder and kidney homogenates collected at sacrifice for Abp1D_RBD_- and Abp2D_RBD_-specific IgG. All animals produced a strong IgG response against both Abp1D_RBD_ and Abp2D_RBD_. This response was enhanced with each subsequent immunization to an AUC > 0.08 at week 12. (Figure 2b). Mice that received Abp1D_RBD_/Abp2D_RBD_ immunizations had significantly (1.5-2 log) lower bacterial titers in bladder tissue and on the catheter surface than mock immunized animals (Figure 2c). Remarkably, the magnitude of the phenotype was significantly increased compared to that observed in convalescent mice (Figure 2d). The degree of antigen-specific IgG in serum and bladder tissue was also much greater in immunized mice compared to convalescent mice (AUC > 0.08 vs. <0.005) (Figure 2e). These data demonstrate that Abp1D_RBD_/Abp2D_RBD_ vaccination both produces greater immunity against two key CAUTI virulence factors than does natural infection and provides a superior level of protection.

### Immunity to Abp2D_RBD_, but not Abp1D_RBD_, is required for protection from *A. baumannii* CAUTI.

**Figure 3:**
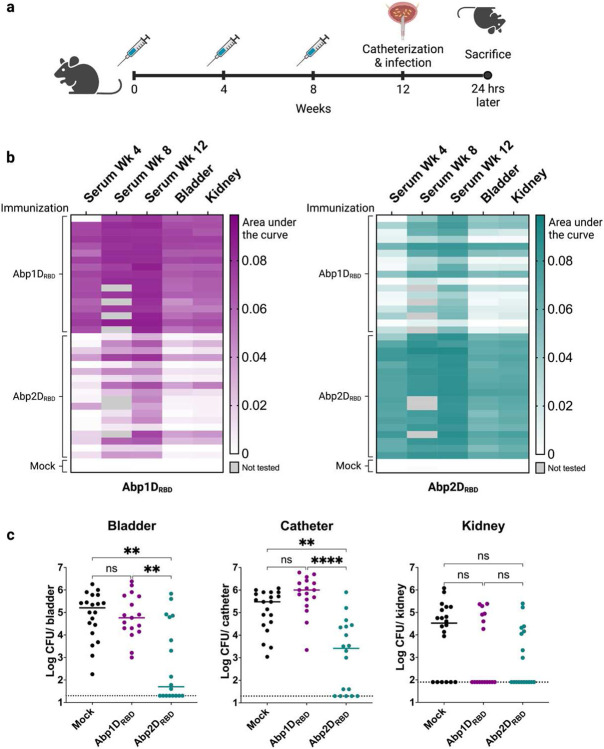
Immunity to Abp2D, not Abp1D, drives protection from CAUTI. a) 6-7 week old C57/Bl6 mice received 3 adjuvanted doses of 50 ug Abp1D_RBD_, Abp2D_RBD_, or buffer alone (mock). Serum was collected at weeks 4 and 8 prior to immunizations, and at week 12 following sacrifice. Four weeks after the third dose, mice were catheterized and challenged with *A. baumannii* strain ACICU and sacrificed 24 hours post-infection. b) Bladder and kidney homogenates and serum from week 4, week 8, and the day of sacrifice were assayed for Abp1D_RBD_ and Abp2D_RBD_-specific IgG by ELISA. Heatmaps were generated by calculating area under the curve for each serum/tissue sample. c) Bacterial titers were enumerated from bladders, catheters, and kidneys. Kruskal-Wallace test with multiple comparisons correction, ****P≤0.0001, **P≤0.005.

As mentioned above, *A. baumannii* deficient in either Abp1D, Abp2D, or both are attenuated in a CAUTI model.^25^ To test whether immunity to both adhesins is required for protection from CAUTI, we immunized mice with each adhesin individually (Figure 3a). We tested the cross-reactivity of the IgG response in immunized animals (Figure 3b) because Abp1D_RBD_ and Abp2D_RBD_ share both structural homology and 70% sequence identity.^25^ Each mouse in the Abp1D_RBD_-immunized group produced a strong Abp1D_RBD_-specific IgG response, but the degree of cross-reactivity with Abp2D_RBD_ varied between animals, with only ~30% displaying strong cross-reactivity. Similarly, each mouse in the Abp2D_RBD_-immunized group produced a strong Abp2D_RBD_-specific IgG response, with strong Abp1D_RBD_ cross-reactivity occurring in ~50% of individuals. Upon catheterization and infection, mice that received Abp2D_RBD_ immunizations were protected from infection, with a statistically significant 2-3 log decrease in bladder and catheter bacterial titers compared to mock-immunized animals (Figure 3c). However, despite high serum levels of Abp1D_RBD_-specific IgG (AUC > 0.08), mice that received Abp1D_RBD_ immunizations were not protected from infection, with bladder and catheter titers equivalent to mock-immunized animals (Figure 3c). This suggests that the lack of protection from CAUTI in Abp1D_RBD_-immunized animals is not due to a lack of immunogenicity. Our studies indicate that immunity to Abp2D_RBD_ alone is both necessary and sufficient for protection from *A. baumannii* CAUTI in this model.

### Abp2D_RBD_ vaccine generates antigen-specific bone marrow plasma cells and splenic memory B cells.

**Figure 4:**
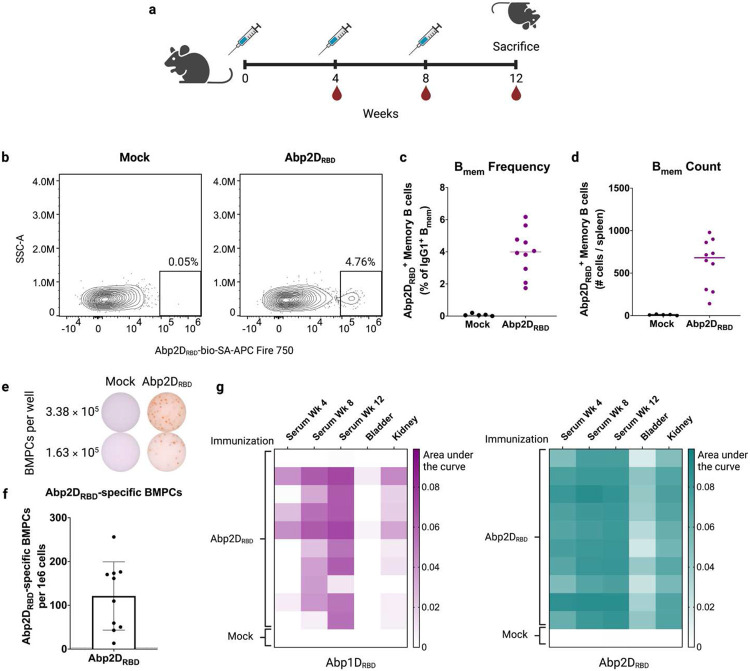
Vaccination with Abp2D generates antigen-specific memory B cells and bone marrow plasma cells. a) 6-7 week old C57/Bl6 mice received 3 adjuvanted doses of 50 ug Abp2D_RBD_ or buffer (mock) and were sacrificed 4 weeks after the 3^rd^ dose. b) Splenic memory B cells (Live/CD4^−^ CD19^+^/IgD^lo^/GL7^−^ CD38^+^/IgG1^+^) were stained with Abp2D_RBD_-biotin and detected with SA-APC-Fire750 via flow cytometry. c) Quantification of Abp2D_RBD_+ splenic memory B cells as % of IgG1+ memory B cells. d) Total Abp2D_RBD_+ memory B cells per spleen. e) Bone marrow was assayed for antigen-specific bone marrow plasma cells via ELISpot. Representative wells from mock-immunized animals and Abp2D_RBD_-immunized animals are shown. f) Quantification of Abp2D_RBD_-specific bone marrow plasma cells in Abp2D_RBD_-immunized animals (n=10). Dotted line indicates the limit of detection (3 cells per 1e6 bone marrow cells). g) Bladder and kidney homogenates and serum from week 4, week 8, and the day of sacrifice were assayed for Abp1D_RBD_- and Abp2D_RBD_-specific IgG by ELISA. Heatmaps were generated by calculating area under the curve for each serum/tissue sample.

A successful vaccine elicits an antibody response that is both high-affinity and long-lasting. We evaluated the immunogenicity of our Abp2D_RBD_ protein subunit vaccine by examining memory B cells and bone marrow plasma cells in immunized animals (Figure 4a). Abp2D_RBD_-specific memory B cells were detectable in the spleens of all immunized animals (Figure 4b-d) by flow cytometry. Antigen-specific memory B cells were defined as lymphocytes/single cells/live/CD4^−^ CD19^+^/IgD^lo^/GL7^−^CD38^+^/IgG1^+^/Abp2D_RBD_-bio-SA-APC-Fire750^+^ (Figure S1). In addition, all immunized animals had detectable Abp2D_RBD_-specific antibody-secreting cells in their bone marrow as assayed by ELISpot (Figure 4e and 4f). We also tested serum and tissue IgG levels in these mice, which were Abp2D_RBD_-vaccinated but did not undergo CAUTI (Figure 4g). Serum and kidney titers were similar to those seen in earlier cohorts. However, bladder IgG levels were reduced (AUC≤0.04) , likely due to a lack of catheterization, which is known to induce significant inflammation and IgG influx.^24^ The presence of antigen-specific bone marrow plasma cells, memory B cells, and high levels of serum IgG indicate that Abp2D_RBD_ vaccination generates all of the hallmarks of immunity of an effective vaccine.

### Passive immunization with serum from Abp2D_RBD_-immunized mice protects naïve mice from CAUTI.

**Figure 5:**
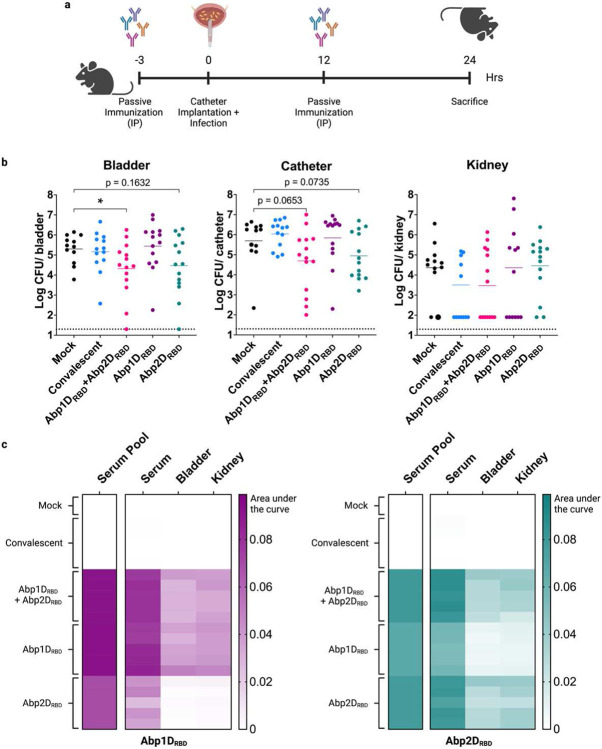
Passive immunization with serum from immunized mice protects naïve mice from CAUTI. a) 6-8 week old C56/Bl6 mice received two 100 ul doses of pooled serum at 3 hours prior and 12 hours after catheterization and infection with *A. baumannii* strain ACICU. Serum was pooled from i) mock immunized and mock infected animals, ii) convalescent animals, or animals immunized with iii) Abp1D_RBD_ + Abp2D_RBD_, iv) Abp1D_RBD_ alone, or v) Abp2D_RBD_ alone. b) Mice were sacrificed 24 hours post-infection and bacterial titers enumerated from bladders, catheters, and kidneys. c) Serum, bladder and kidney homogenates from infected mice were assayed for Abp1D_RBD_- and Abp2D_RBD_-specific IgG by ELISA. The serum pools used for immunizations were also tested and are shown on the left of each heatmap. Heatmaps were generated by calculating the area under the curve. Mann-Whitney U-test, *P≤0.05.

If the immunity conferred by the Abp2D_RBD_ vaccine is due to serum IgG, then transferring IgG from immunized animals to naïve animals should also provide protection from a CAUTI challenge. To test the degree to which immunity conferred by the Abp2D_RBD_ vaccine is antibody-mediated, we administered serum pooled from 5 groups of mice: i) mock immunized; ii) convalescent; iii) Abp1D_RBD_ + Abp2D_RBD_ immunized; iv) Abp1D_RBD_-immunized; and v) Abp2D_RBD_-immunized (Figure 5a). Mice that received serum from Abp1D_RBD_ + Abp2D_RBD_ immunized animals had a statistically significant reduction of ~1 log in bladder bacterial titers (P<0.05), while mice receiving serum from convalescent or Abp1D_RBD_-immunized mice were not protected (Figure 5b). Mice that received serum from Abp2D_RBD_-immunized animals had a 1 log reduction in bladder bacterial titers trending towards significance (P=0.1632). The smaller effect size compared to vaccination is not unexpected, since passively immunized mice have a much lower concentration of adhesin-specific antibodies in their bladder and kidney tissues (Figure 5c). These data demonstrate that humoral rather than cellular immunity is the likely driver of protection in our vaccine model.

## Discussion

Catheter-associated urinary tract infections are the second most common cause of healthcare-associated infections. Although *A. baumannii* causes a small percentage of all CAUTIs, these infections are often multi-drug resistant and frequently life-threatening for affected patients, leading the CDC to label *A. baumannii* as a “pathogen of urgent concern.”^13^ Thus, there is a critical need to develop novel antimicrobial strategies to combat this infection. Here we demonstrate that a vaccine targeting the interaction between *A. baumannii* and its ligand provides protection from CAUTI. Our Abp2D_RBD_ vaccine elicits many features of a successful immune response including memory B cells, bone marrow plasma cells, and high levels of serum and tissue IgG. While several vaccine strategies have been attempted for *A. baumannii* with mixed results,^34^ to our knowledge this is the first report of an *A. baumannii* vaccine that is effective in preventing CAUTI pathogenesis in a mouse model.

Other adhesin-based vaccines for the treatment and prevention of urinary tract infections have been reported in the literature. A vaccine against the *E. coli* FimH pilus adhesin, which has been shown to be critical in interactions initiating and perpetuating *E. coli* cystitis, has recently completed a Phase Ia/Ib human clinical trial. This trial showed that the vaccine reduced the incidence of recurrent UTI by more than 75% in vaccinated patients.^35^

Analogous to COVID vaccines that target the SPIKE protein, our strategy is to neutralize the adhesin that *Acinetobacter* uses for binding fibrinogen-coated catheters, leading to infection. Both Abp1 and Abp2 pili are capable of binding to fibrinogen, and both pili play a role in CAUTI.^25^ Thus, we expected that immunity to both Abp1D and Abp2D would be required in order to fully “neutralize” the bacteria and prevent adhesion to the catheter. It was therefore surprising that immunity to Abp1D_RBD_ proved to be unnecessary for protection from CAUTI. Although mice immunized with Abp1D_RBD_ generated high serum levels of Abp1D_RBD_-specific IgG, including IgG capable of cross-reacting with Abp2D_RBD_, there was no protective effect. Structural studies of Abp1D_RBD_ and Abp2D_RBD_ provide a possible explanation for this observation. While the two adhesins share a great deal of structural similarly, the anterior loop of the binding pocket is considerably more flexible in Abp1D_RBD_ than in Abp2D_RBD_.^25^ Because of this flexibility Abp1D_RBD_ can adopt either a “closed” (lower affinity) or “open” (higher affinity) conformation. An antibody response generated against the “closed” conformation is unlikely to recognize the binding pocket and therefore unlikely to functionally inhibit binding to a fibrinogen coated catheter. Conversely, the anterior loop of Abp2D_RBD_ is more rigidly positioned in an open conformation and therefore presents a more reliably accessible antibody epitope.^25^ The differences in both conformation and flexibility between the binding pockets of the two proteins may explain why Abp2D_RBD_ immunization is more protective than immunization with Abp1D_RBD_. Other explanations for the difference between Abp1D_RBD_ and Abp2D_RBD_ vaccines may include changes in pilus expression, variations in epitope availability, or other differential factors.

Although we demonstrated that our Abp2D_RBD_ vaccine produces a robust antigen-specific IgG response and that this immunity is transferable through serum, one limitation of this study is that we are unable to identify which specific properties of the antibody response are providing the protection from challenge. We initially hypothesized that protective antibodies would “neutralize” bacteria by physically blocking the interaction between Abp2D and its ligand, fibrinogen, to reduce catheter bacterial colonization and thus prevent bladder infection. Vaccinated mice demonstrate a reduction in catheter bacterial titers, including several individual animals that completely excluded *A. baumannii* colonization of the catheter, so this mechanism of action is plausible. However, antibodies can also promote infection clearance through other mechanisms such as opsonization and complement activation. Future studies will attempt to establish which properties of the Abp2D_RBD_ antibody response are most essential for protection and optimize Abp2D_RBD_ immunizations to maximize efficacy.

Vaccines have an important role to play in reducing the incidence of disease and decreasing opportunities for natural selection of antibiotic-resistance. However, it is difficult to predict which patients may develop an *A. baumannii* infection and therefore challenging to identify who would most benefit from vaccination. One potential patient cohort is chronically catheterized patients. CAUTI risk increases by 3-7% for each day of catheterization, leading to an almost 100% probability of CAUTI in patients who remain catheterized over the long term.^3^ Once established, CAUTI can be highly recurrent in spite of repeated antibiotic administration. Thus, chronically catheterized patients may be good candidates for prophylactic vaccination against CAUTI pathogens such as *A. baumannii*. In addition, given that *A. baumannii* can establish intracellular reservoirs within bladder epithelial cells, patients with a history of *A. baumannii* cystitis may benefit from vaccination to prevent recurrence.^9^ However, perhaps the greatest potential benefit of an *A. baumannii* vaccine lies in the developing world. The highest relative burden of deaths associated with antibiotic-resistant *A. baumannii* occurs in low and middle income countries.^12^ Health centers in Somalia and Kuwait report that *A. baumannii* accounts for up to 25% of CAUTIs in their facilities.^10,11^ In this setting, the storage conditions required for a protein subunit vaccine (e.g., simple refrigeration) present an advantage over more modern vaccine modalities.^27^

Our findings highlight how basic research into microbial pathogenesis, such as the identification of pili implicated in CAUTI, can be translated into effective, antibiotic-sparing therapeutics. An Abp2D_RBD_ vaccine has the potential to reduce *A. baumannii* CAUTI incidence in vulnerable patient populations and has an important role to play in the fight against antimicrobial resistant infections.

## Methods

### General bacteriology.

Bacterial stocks were maintained as glycerol stocks at −80°C. Strains were streaked on LB-agar plates and incubated at 37°C for 14-18 hours, at which time colonies were selected and used to inoculate liquid low-salt LB media (10 g tryptone, 5 g NaCl, and 5 g yeast extract per L). All bacterial cultures used in this study were grown statically at 37°C for 24 hours followed by 1:1000 dilution and subculture for an additional 18-20 hours. Bacteria were spun down at 3000xg, washed 1x in PBS, resuspended at the specified OD_600_, and kept on ice until use. *A. baumannii* strain ACICU, representative of global clone 2,^36^ was used for all experiments described in this study.

### Protein purification and labeling.

Protein was expressed and labeled as previously described.^24^ Briefly, cells were harvested in a large-scale fermenter format from C600 containing expression plasmids, grown to mid-logarithmic phase, and induced with 0.1mM IPTG for 1 h. The culture was subsequently harvested, and the periplasm isolated generally as described previously.^35^ RBD protein constructs and mutants were purified by cobalt affinity chromatography, eluted at ~150mM imidazole with a gradient of 1xPBS to 1xPBS/300mM imidazole. Protein-containing fractions were pooled and run on a Source 15S (Tm GE) cation-exchange column and eluted at 30mM NaCl with a gradient of 20mM MES pH 5.7 to 20mM MES pH 5.7/200nM NaCl. Purified protein was subsequently dialyzed or buffer exchanged into 20 mM MES pH 5.8 + 50 mM NaCl. Where required, protein was biotinylated using the EZ-Link NHS-PEG4 biotinylation reagent (Thermo Scientific) and diluted in H_2_O to 100 mM. Protein was either dialyzed or buffer exchanged into 1× PBS. Biotinylation reagent was added at a 20 fold molar excess for 2 h at 4 °C under rocking. Biotinylated protein was subsequently dialyzed into PBS, removing the excess biotin reagent.

### Murine immunizations.

All immunizations were prepared by mixing 50 μg/mouse of Abp1D_RBD_ or Abp2D_RBD_ 1:1 by volume with Addavax, a squalene oil-in-water adjuvant (Invivogen) to a total volume of 50 μL/mouse. Mock immunizations were prepared by mixing buffer 1:1 with Addavax. C57Bl/6 mice were obtained from Charles River Laboratories and were 7-9 weeks old at the first immunization. Mice were immunized intramuscularly in the hind flank at weeks 0, 4, and 8, for a total of 3 immunizations of 50 μg/protein each. For dual immunization experiments, each mouse received 50 μg of Abp1D_RBD_ in the left hind flank and 50 μg of Abp2D_RBD_ in the right hind flank at each time point. Blood was collected at weeks 4 and 8 by submandibular or submental collection prior to the administration of the immunization.

### Murine CAUTI model.

Mice were catheterized and infected as described previously.^8^ Briefly, mice were anesthetized with 4% isoflurane/0.8% oxygen by inhalation. A short piece of silicone tubing (4-5 mm) was transurethrally inserted into the bladder and immediately followed by 2 doses of 2x10^8^ CFUs of *A. baumannii* strain ACICU in 50 μl of PBS (OD600 ~13). 24 hours after infection, mice were anaesthetized and humanely sacrificed by cervical dislocation. Blood was collected from the inferior vena cava, allowed to clot for 30 minutes at room temperature, and spun down to remove red blood cells and clotting factors. Serum was removed to a new tube and frozen at −20°C until analysis. Catheters were removed from bladders, placed into 1 mL of sterile PBS, and processed by vortexing for 30 seconds, sonicating for 10 minutes, and vortexing for an additional 30 seconds to remove biofilm and bacteria from the catheter surface. Bladders and kidney pairs were both placed into tubes containing sterile stainless steel beads and sterile PBS (1 ml for bladders, 800 μl for kidney pairs) and homogenized at 4°C using the MP Biomedical Fastprep-24 homogenizer. The homogenization settings used were 1 min shaking at 4 m/s, 5 min of rest, followed by an additional 1 min of shaking. Bladder, kidney, and catheter samples were serially diluted and plated on selective media (LB + 100 μg/L Ampicillin). Plates were incubated at 37°C for 12-16 hours and bacterial cfus enumerated. Remaining bladder and kidney homogenates were frozen at −20°C for additional analyses.

### Convalescent infection model.

7-9 week old C57Bl/6 mice were catheterized and infected as described above. One group of mice received 2 doses of 2x10^8^ cfus of ACICU, and the other group received sterile PBS. Urines were collected at days 3, 7, 10, 14, and weekly thereafter. Blood was collected by submandibular or submental collection at weeks 3, 5, and 8, and at time of sacrifice. At week 5, mice were treated with 1 g/L Apramycin for 10 days to clear bacteriuria. At week 8, mice were again catheterized and infected, then sacrificed at 24 hours post infection as described above.

### ELISAs.

All ELISAs were performed using Grenier Microlon high-binding plates (Grenier Bio-One #655085). Plates were coated with 100 μl of 1 μg/ml Abp1D_RBD_, Abp2D_RBD_, or *E. faecalis* EbpA^NTD^ (used as a negative control for anti-HIS antibodies) in PBS and incubated overnight at 4°C. The following morning plates were washed 1x with 200 μl PBS containing 0.05% Tween-20 (PBS-T). Plates were blocked with 300 μl of PBS-T containing 10% fetal bovine serum (P10) for 1.5 hours at room temperature. Serum, bladder, and kidney homogenates were diluted 1:30 into 75 μl P10 and then serially diluted 1:3 and incubated for 1 hour at room temperature. Plates were washed 3x in PBS-T. Goat anti-mouse-IgG-HRP secondary antibody (Southern Biotech Cat# 1030-05) was diluted 1:1000 in P10 and 100 μl added to each well and incubated for 1 hour at room temperature in the dark. Plates were washed 3x with PBS-T followed by 3x with PBS, developed with 100 μl developing reagent and quenched with 100 μl of 1 M HCl. Developing reagent consists of 10 ml phosphate-citrate buffer (Sigma Cat# P4809), 4 mg o-Phenylenediamine dihydrochloride (Sigma Cat#P8787), and 33 μl 3% H_2_O_2_ per plate. Plates were read using the BioTek ELx800 plate reader on the OD490 setting. Graphpad Prism 9 was used to calculate area under the curve for each sample. AUCs were baseline corrected by subtracting the AUC binding to the negative control protein, EbpA^NTD^,^23^ which contains the same 6x His tag used to purify Abp1D_RBD_ and Abp2D_RBD_ but is otherwise structurally unique.

### ELISpot.

PVDF-membrane plates (Millipore Sigma #MSIPN4W50) were prepared by activating with 50 μl of 35% ethanol for 30 seconds followed by washing 3x with PBS. Plates were coated with 100 μl of 5 μg/ml Abp2D_RBD_ or anti-mouse IgG (positive control) in PBS and incubated overnight at 4°C. The next morning, plates were washed 3x with PBD + 0.05% Tween-20 (PBS-T) and blocked with 200 μl of RPMI media containing 10% fetal bovine serum (R10) for 2 hours at 37°C and 5% CO_2_. Mice that were immunized as described above were sacrificed 4 weeks after the third immunization. Bone marrow was collected from both femurs into R10, washed, and resuspended to a concentration of 1x10^7^ cells/ml. 5x10^5^ cells were added to the first well and serially diluted. Plates were incubated for 4 hours at 37°C and 5% CO_2_. Plates were washed 1x with PBS and 3x with PBS-T. 100 μl of biotinylated anti-mouse IgG (Southern Biotech Cat#1030-08) diluted 1:1000 in PBS containing 2% fetal bovine serum and 2mM EDTA was added to the plate and incubated overnight at 4°C. The next day, plates were washed 3x with PBS-T. HRP-conjugated streptavidin (Jackson Immunoresearch Cat#016-030-084) was diluted 1:5000 in PBS + 2%FBS/2mM EDTA, 100 μl added to each well, and the plates incubated for 1.5 hours at room temperature in the dark. Plates were washed 3x with PBS-T followed by 1x with PBS. Developing solution was prepared by diluting 3 mg of 3-amino-9-ethylcarbazole in 10 ml of 0.1 M sodium acetate buffer, pH 5.0 and syringe filtering through a 0.45 PVDF membrane. Just prior to use, 100 μl of 3% H_2_O_2_ was added to the mixture. 100 μl of developing solution was added to each well and allowed to incubate until spots were visible, ~5 minutes. Developing solution was removed and plates washed under DI water to halt the reaction. Plates were dried overnight at room temperature and imaged using the CTL ImmunoSpot imager (Cellular Technology Limited). Spots were counted using the CTL ImmunoSpot automatic counting program with default parameters.

### Flow cytometry.

Mice that were immunized as described above were sacrificed 4 weeks after the third immunization. Spleens were collected into RPMI containing 2% fetal bovine serum and manually homogenized using the back of a syringe plunger. Cells were filtered through 75 um mesh, washed 1x, and counted. 2x10^7^ splenocytes were stained for flow cytometry. All washes for the staining process were performed in PBS containing 2% fetal bovine serum and 2 mM EDTA. Cells were incubated with CD16/32 (Biolegend Cat# 101302) and 5.875 μg/ml of biotinylated Abp2D_RBD_ for 10 minutes, then washed 3x. A cocktail containing the following antibodies was prepared in BD Brilliant Staining Buffer (BD Cat. # 563794), all sourced from BioLegend unless otherwise indicated : Zombie NIR (Cat#423105), CD19-BV750 (Cat#115561), CD4-BV570 (Cat#100542), IgD-BV711 (Cat#405731), IgM-BV605 (Cat#406523), IgG1-BV510 (Cat#406621), Fas-PE (BD Cat# 554258), GL7-PcpCy5.5 (Cat# 144610), CD38-PE-Cy7 (Cat#102718), CD138-BV421 (Cat#142508), and streptavidin-APC-Fire-750 (Cat#405250). Invitrogen UltraComp eBeads were used for single colors. Flow cytometry data was collected using the Cytek Aurora with 4 laser 16V-14B-10YG-8R configuration and processed on FlowJo10 for Mac.

### Passive immunization model.

Serum was collected at the time of sacrifice for all immunized and convalescent animals described above, and this serum was used for passive immunization experiments. 200 μl of serum from each individual within a group was combined to form the serum pools. Serum pools were sterile filtered and frozen in aliquots at −20°C until use. Five pools were prepared: i) Mock immunized/Mock infected, ii) Convalescent, iii) Abp1D_RBD_ + Abp2D_RBD_ immunized, iv) Abp1D_RBD_ immunized, v) Abp2D_RBD_ immunized. Naïve, 7-9 week old C56Bl/6 mice received 1 dose of 100 μl pooled serum 3 hours prior to catheterization and infection as described above. Mice received a second dose of 100 μl pooled serum 12 hours post infection. Mice were sacrificed at 24 hpi and tissue titers enumerated as described above.

### Statistical analysis.

All statistical tests were performed using built-in statistical functions of GraphPad Prism 9. All data analyzed for statistical significance (e.g., bacterial titer data) were nonparametric. The Mann-Whitney *U* test was used for comparisons of 2 groups. The Kruskal-Wallis test with multiple comparisons correction was used for comparisons of 3 or more groups.

## Supplementary Material

Supplement 1
