# Supplementary material for: Vaccination with Acinetobacter baumannii adhesin Abp2D provides protection against catheter-associated urinary tract infection
Source: Res Sq. 2023 Aug 10:rs.3.rs-3213777. Preprint. [Version 1] doi: 10.21203/rs.3.rs-3213777/v1 (PMC10441454; doi:10.21203/rs.3.rs-3213777/v1)
Supplement: Supplement 1 [file NIHPPrs3213777v1-supplement-1.pdf]

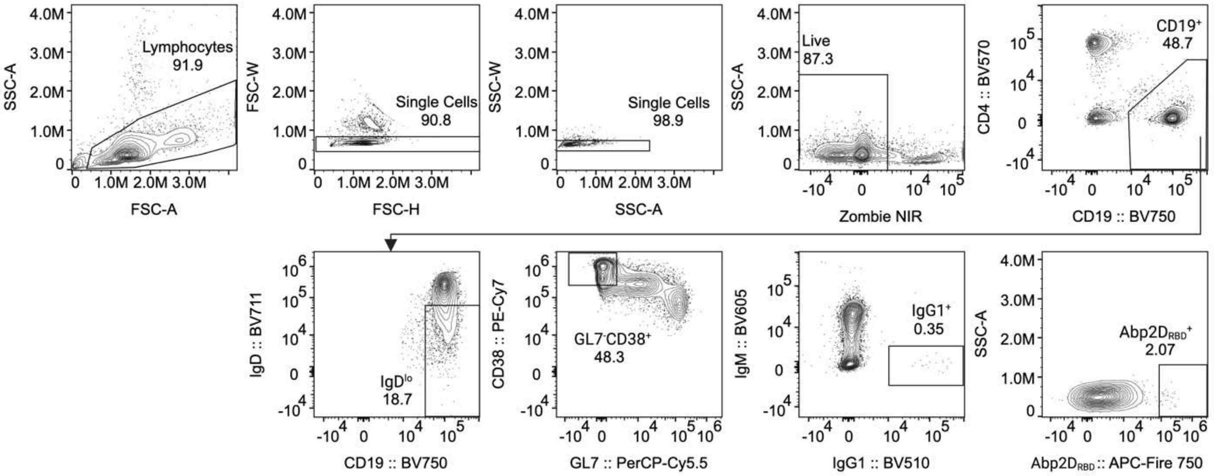

Supplemental Figure 1: Flow cytometry gating strategy for Abp2D+ splenic memory B cells. Splenocytes were stained and gated on lymphocytes/single cells/live/$CD4^{-}$ $CD19^{+}$/IgD^{lo}/$GL7^{-}$ $CD38^{+}$/$IgG1^{+}$/$Abp2D_{RBD}^{+}$.
